# Patient-Centered Endodontic Outcomes: A Narrative Review

**Published:** 2013-10-07

**Authors:** Reza Hamedy, Bita Shakiba, Sara Fayazi, Jacklyn G Pak, Shane N White

**Affiliations:** a UCLA School of Dentistry, CA, USA; b Section of Endodontics, UCLA School of Dentistry, Los Angeles, CA, USA

**Keywords:** Cost and Cost Analysis, Dental Anxiety, Fear, Patient-Centered Care, Personal Satisfaction, Quality of Life, Root Canal Therapy

## Abstract

**Introduction:**

Root canal treatment (RCT) success criteria inform us of the path to bony healing and of prognostic factors, but tell little about how the patient perceives, feels, or values RCT. Patients choose, undergo, and pay for RCT, they live with the result, and inform their community. The purpose of this narrative review was to appraise patient-centered outcomes of initial non-surgical RCT and nonsurgical retreatment, in adults.

**Materials and Methods:**

Patient-centered RCT outcome themes were identified in the extant literature: quality of life, satisfaction, anxiety, fear, pain, tooth survival and cost. Narrative review was applied because the disparate themes and data were unsuited to systematic review or meta-analysis.

**Results:**

Application of the Oral Health Impact Profile (OHIP) demonstrated that disease of pulpal origin affects quality of life with moderate severity, primarily through physical pain and psychological discomfort, and that RCT results in broad improvement of quality of life. Satisfaction with RCT is extremely high, but cost is the primary reason for dissatisfaction. Anxiety and fear affect RCT patients, profoundly influencing their behaviors, including treatment avoidance, and their pain experience. Fear of pain is “fair” to “very much” prior to RCT. Pain is widely feared, disliked, and remembered; however, disease of pulpal origin generally produces moderate, but not severe pain. RCT causes a dramatic decrease in pain prevalence and severity over the week following treatment. Survival rates of teeth after RCT are very high; complication rates are low. Cost is a barrier to RCT, but initial costs, lifetime costs, cost effectiveness, cost utility, and cost benefit all compare extremely well to the alternatives involving replacement using implants or fixed prostheses.

**Conclusion:**

Dentists must strive to reduce anxiety, fear, experienced and remembered pain, and to accurately inform and educate their patients with respect to technical, practical and psychosocial aspects of RCT.

## 1. Introduction

Endodontic outcomes have been long and widely studied in terms of root canal treatment (RCT) case success. Instruments such as the Strindberg Criteria or Orstavik’s Periapical Index have been widely used to measure treatment outcomes. These instruments are extremely helpful in studying prognostic indicators and in measuring the long and irregular pathway towards radiographic bony healing (1-3). Even though Strindberg’s criteria include reference to patient symptoms, such prognostic instruments tell us little about how the patient perceives, feels, or values the treatment. It is the patient who chooses the RCT, undergoes it, pays for it, lives with the experience, and informs their family, friends and community.

Patients’ pretreatment decisions and post treatment satisfaction may be strongly influenced by social, psychological, and behavioral dimensions including knowledge, beliefs, attitudes, preferences and behaviors (4). Patients may know little about endodontic pathophysiology, but are likely to be highly sensitized to treatment-related fear, anxiety and pain; concerned about cost, and whether the treated tooth fulfills their functional and esthetic expectations. Although it can be argued that patient-centered outcomes may be less objective than radiographic indices, the field of psychometry is well developed and includes such properties as validity, reliability and responsiveness. Patient-centered outcomes may complement radiographic indices. Some patient-centered outcome measures are patient-reported, but others are measured externally (5). Patient-centered outcome measures provide feasible and appropriate methods for addressing patients’ concerns (6). Clinicians’ perspectives of oral health are limited; however, the patient can tell us how root canal treatment affects their physical, psychological and social function, i.e. their quality of life (7).

The study of endodontic patient-centered outcomes is a rapidly expanding area, but one that has not yet received broad review or overall synthesis in the dental literature. The purpose of this narrative review was to appraise patient-centered outcomes of initial non-surgical root canal treatment, initial treatment and nonsurgical retreatment, in adults.

## 2. Material and Methods

Patient-centered RCT outcome themes were identified in the extant literature: quality of life, satisfaction, anxiety, fear, pain, tooth survival and cost.

Narrative review, rather than systematic review, was chosen because the studied topic was broad-reaching, contained disparate data types, was heterogeneous, and identified data was not conducive to meta-analysis. Wherever possible, higher forms of clinical evidence, such as systematic reviews, were referenced. For convenience, we have grouped patient-centered outcomes into the common themes represented in the extant literature.

### 2.1. Quality of Life and Satisfaction

Quality of life is concerned with the degree to which a person enjoys the important possibilities of life (8). Factors measured in the Oral Health Impact Profile (OHIP) (9) questionnaire including: functional limitation, pain, psychologic discomfort, physical disability, psychologic disability and social disability have been studied with respect to RCT (7, 10-12).

Dugas et al., studied the patients who had received RCT within the last 2 years, using a modified seven-question version of OHIP, emphasizing on diseases of pulpal origin (10).

Physical pain, psychological discomfort, and psychological disability highly influenced postoperative quality of life; social disability and handicap moderately influenced postoperative quality of life; functional limitation had little influence on postoperative quality of life (10). Physical pain and psychological discomfort had high impacts; psychological disability had a moderate impact; social disability, physical disability, handicap, and functional disability had low impacts (10). Physical pain and psychological disability had high percentages of improvement after RCT; social disability and handicap, and physical disability had moderate percentages of improvement; psychological discomfort and functional limitation had the lowest percentage of improvement (10)

In two subcategories, the ability to perform usual jobs (social disability) and temperature sensitivity (physical pain), RCT which was provided by endodontists produced significantly more improvement than by generalists (10). For the same subcategories patients with high Orstavik Periapical Indices also experienced more improvement than others. Dugas et al. also measured patient satisfaction using a 10-point semantic scale; general satisfaction ratings were high. The vast majority of subjects reported satisfaction with their decision to have RCT rather than extraction. Interestingly, satisfaction improved significantly more in a large city sample than in a small-city sample. Cost was by far the single greatest cause of dissatisfaction with RCT, but time, pain during RCT, pain after RCT and poor esthetics were also reported (10).

Hamasha and Hatiwsh used the OHIP questionnaire, as used by Dugas et al. with 17 questions (10), before and 2 weeks after RCT (12). Before treatment, physical pain had a high prevalence of impact; psychological discomfort had a moderate prevalence, and the other fields had low prevalence of impact. They found marked improvement in physical pain, psychological discomfort, physical disability, psychological disability, social disability; and substantial improvement in functional limitation and handicap after RCT. No difference in quality of life was found with respect to provider, specialist, graduate student, or generalist. Hamasha and Hatiwsh also studied satisfaction 2 weeks after treatment using the semantic differential scale, previously used by Dugas et al. (10) General satisfaction was extremely high, 8.6 on a 10-point scale. Patients ranked their satisfaction from highest to lowest as: intraoperative pain, pleasantness, general satisfaction, chewing ability, time involved, cost, and postoperative esthetics. Overall satisfaction was significantly influenced by provider (specialist, generalist or student) and by income level.

Gatten et al. used a 14-question shortened version of the OHIP questionnaire delivered at least one year after RCT or implant and coronal restoration (9, 11). They reported some comparable findings; physical pain and psychological discomfort had the highest prevalence. However, unlike Dugas et al., they found a significant gender difference in severity scores for psychological disability. Focus group discussions revealed frequently mentioned themes: it was important to keep their teeth, teeth are part of overall health; cost was high, but insurance helped; the additional cost of the crown caused surprise; tight contacts of new crowns caused trouble flossing; those receiving anterior treatment felt better esthetically; family and peers strongly influence received treatment; those with preoperative pain appreciated relief during and after treatment; minimal pain was reported during treatment; less pain was experienced than expected; the worst pain was from injection; a few patients reported sensitivity that was not painful; patients complained of opening their mouths for a long time; bite blocks helped but were still uncomfortable; jaws were sore or hard to close after the treatment; the time needed for crown completion after RCT was a concern; patients attend the dentist more regularly after treatment, but might not attend for recall if not in pain; the tooth lacked temperature sensitivity after treatment; the tooth was maintained in the same way as other teeth; peace of mind that infection is gone; saving a tooth in of itself had little effect on esthetics, but was considerably better esthetically than losing the tooth; and overall satisfaction was high, even if mishaps occurred. Overall, both RCT and implant patients were pleased with the treatment received and expressed a clear message to save their natural dentition whenever possible.

Liu et al. used the 14-question shortened version of the OHIP questionnaire (9) to compare quality of life between patients scheduled for RCT and patients receiving periodontal maintenance (7). The fields of physical pain, psychological discomfort, physical disability, psychological disability had a moderate impact; whereas, the fields of social disability, handicap and functional limitation had low impact. Patients awaiting RCT had overall summary scores approximately 1.7 times higher than those receiving periodontal maintenance.

Yu et al. studied painful exacerbations of persistent periapical lesions using a modified Oral Impacts on Daily Performances (OIDP) quality of life questionnaire (13). The vast majority of patients reported only low levels of impact, but a small minority reported substantial impact, primarily in the areas of eating, enjoying food, and tooth cleaning (14).

Lobb et al. studied patients’ perceptions of their RCT. They reported that the majority of patients who received endodontic treatment would undergo this treatment again if their dentist recommended it; for those few who would not, pain and expense appeared to be the greatest deterrents (15).

Gorduysus and Gorduysus studied expected and experienced pain, satisfaction of RCT, and economics (16). Almost all patients expected that RCT would save their teeth. A small minority, 15%, who initially favored extraction over RCT markedly decreased to 2.5% post treatment. The vast majority was satisfied with their RCT, would chose to have RCT again and recommend it to others. Pre-treatment expectation findings suggested that dentists need to be better at providing RCT outcomes.

Jimena studied geriatric patient attitudes and RCT satisfaction; patients generally reported a positive attitude to RCT, reporting relief of pain, better appreciation of food and improved self-esteem (17).

In summary, disease of pulpal origin affects quality of life with moderate severity, primarily through physical pain and psychological discomfort. Provision of RCT resulted in broad improvement of quality of life, especially in physical pain, psychological discomfort, psychological disability, and social disability. Some provider and patient differences were noted. Satisfaction with RCT was extremely high but cost was the primary reason for dissatisfaction.

### 2.2. Anxiety and Fear

Anxiety and fear are certainly felt by RCT patients; this may be expressed in a variety of different ways ranging from physiologic responses such as hyperventilation or fainting, to simple verbal expression, and to silence or loquaciousness (18). Patients may also cry, use facial expressions, or body language, such as clenching their fists, or gripping the arm of a dental chair. Dentists must be attentive to all expressions of anxiety and fear and appreciate patient’s perspectives whether they appear rational or not.

Dental anxiety, fear and phobia are known to profoundly influence patients’ behaviors and felt experiences (19-22). Fearful patients are more likely to experience and remember more pain (19, 21). They also tend to avoid necessary treatment, perpetuating a vicious cycle of dental fear and avoidance (20, 22-26). Likewise anxiety can produce a vicious cycle of overestimation of pain and increased anxiety (27). Reasons for anxiety include feelings of vulnerability, danger, lack of control, unpredictability, and expectation of pain (19, 21, 23, 28). Higher levels of educational attainment are associated with reduced dental fear and with reduced avoidance of dental treatment (21, 23).

Although anxiety has been well studied with respect to the general field of dentistry, less has been reported specifically concerning RCT. This is surprising because RCT appears to carry a special stigma beyond all other dental disciplines and feature prominently in patients’ life stories (29).

There is no doubt that RCT can increase patients’ physiologic and psychological stress levels (30). Patients scheduled to undergo RCT experience “fair” to “very much” fear of pain, or 3-4 on a 5-point scale (28). Experienced pain during RCT is correlated to the level of anticipated anxiety (27, 30). Women tend to experience more RCT associated anxiety and anticipate more pain than men, but women may not actually experience more pain than men (18, 31, 32). Younger adults anticipate and experience higher pain levels [32]; they may also experience more anxiety (31).

Physiologic stress peaks early in a RCT appointment, around the time of local anesthesia delivery and initial instrumentation (30, 33). Patients ranked the following RCT steps from least to most anxiety producing as: electric pulp testing, rubber dam, appointment length, multiple radiographs, rubber dam clamp placement, X-ray film placement, access opening, percussing a sore tooth, sensing files, local anesthesia injection (18).

Patients may avoid RCT due to anxiety and fear of pain, resulting in treatment avoidance and eventual tooth loss through extraction (34). Half of patients reported no change in fear after RCT, but 44% reported less fear after RCT, and only 6% reported more fear after RCT (18). Prior experience of RCT tends to decrease anxiety; experience may counteract negative hearsay (18, 29). Interestingly, negative hearsay increases RCT associated anxiety, but prior negative RCT experiences do not increase anxiety (29).

Accurately informing patients about pain associated with RCT reduces fear of pain (27). Fortunately, patients almost unanimously would choose to have RCT again to save a tooth (18). It has been suggested that dentists be trained in behavioral management, nitrous oxide, and conscious intravenous sedation so as to improve access to RCT care (35, 36). Dentists and their assistants must carefully inform and educate their patients, and do all that they can to reduce anxiety and fear (27, 35-37).

### 2.3. Pain

Pain is widely feared and disliked by the public (24, 29, 38, 39). Patients describe toothache pain as intense, throbbing, piercing, miserable, and unbearable. Toothache has a profound behavioral impact affecting mood, ability to perform normal activities, sleep, job, and social activity (38). In addition to attending the dentist, a wide variety of self-care is used to address toothache, including over the counter medicines, over the counter dental products, prescription medicines (including those prescribed for others), a wide variety of home or folk remedies, and prayer (38). Cost, time availability, fear of dentists, fear of needles, fear of pain, and anxiety that a dentist may find other problems are all barriers to care of toothache (38). However, patients overwhelmingly identify receiving care from a dentist as the preferable option for pain relief (38). Toothache can be relieved by extraction or RCT.

It appears that patients often remember a connection between pain and RCT, rather than a connection between pain and extant disease of pulpal origin or its predominant cause, caries. Unfortunately, RCT appears less frequently remembered for relief of pain. However, RCT definitely causes a dramatic decrease in pain (39, 40). Pretreatment RCT associated pain prevalence is high, but drops moderately within one day of treatment, and to minimal levels in a week (39). Pretreatment RCT associated pain severity is generally moderate, drops substantially within 1 day of treatment, and continues to drop to minimal levels in a week (39). Perhaps, patients fail to associate pain relief with RCT because the relief occurs gradually in the days following RCT, rather than instantaneously. Preoperative anxiety and fear levels may be high; however, pain of endodontic origin is generally moderate, contrary to popular folklore, and RCT decreases pain.

Pain is frequently experienced during RCT, but generally only at low levels of severity (39, 41). Pain during RCT is also usually less than anticipated (11, 16, 32). Pretreatment diagnoses such as irreversible pulpitis and acute apical periodontitis have been associated with increased intraoperative pain (41). Intraoperative pain prevalence tends to increase after 45 min of treatment (41), presumably as initial anesthesia wears off. Dentists must be vigilant and supplemental anesthesia must often be provided.

Many studies have attempted to identify predictive factors for post treatment pain. However, results have often been unclear, inconsistent, or lacking obvious mechanistic cause (42, 43). It is not unreasonable that patients with more pretreatment pain may experience more post treatment pain (39, 44).

The influence of single versus multiple appointments on post treatment pain has been widely studied, subjected to systematic reviews and meta-analysis. Although patients undergoing single-visit RCT reported a higher frequency of pain medication use, compelling evidence for a difference is lacking (45, 46). Likewise, it appears that post-treatment pain does not differ between initial RCT and retreatment (44).

Flare ups resulting in pain, swelling, and unscheduled attendance following a RCT appointment generally have a low incidence (14, 33, 47-50). Flare ups are most likely of bacterial origin, and often occur a day or two after an otherwise uneventful RCT appointment (51). Many precipitating factors have been studied; however sufficient evidence to identify prognostic factors is absent (49).

Long term persistent pain following RCT is rare (52), but of obvious importance to those suffering. Such persistent pain could be ascribed to inadequate healing following RCT, or to non-endodontic sources of pain. Nonodontogenic pain may represent up to half of all cases of persistent pain (53). These findings emphasize the importance of careful and accurate pretreatment diagnosis and post treatment follow up.

Pain of pulpal origin is best managed by RCT and non-steroidal anti-inflammatory drugs; antibiotics should not be prescribed to treat pain or the expectation of pain (51, 54). Dentists must strive to minimize pain experiences during and after RCT as well as fear and anxiety.

### 2.4. Tooth Survival

The survival of a treated tooth is of obvious importance to the patient. Little in life is certain, but patients may reasonably expect a high probability for long-term retention of their treated teeth (16). Long-term survival rates for endodontically treated teeth are very high, typically over 90% (55-58). Excellent systematic reviews have been published by Torabinejad et al., Iqbal and Kim, and Ng et al (57-59). Torabinejad et al. showed that both RCT and single-tooth implants resulted in very high long term, 6 plus year, weighted survival rates of 97%, compared to only 80% for 3 or 4 unit fixed dental prostheses (57). Interestingly, the absence of preoperative pain has been associated with a decreased risk of tooth loss after RCT (60).

The endodontic literature is distinguished by studies with unusually large sample size. These include: Lazarski et al., 94% functional survival for 44,613 cases at 3.5 years in the USA; Salehrabi and Rotstein 97% survival for 1.1 million patients at 8 years in the USA; and Chen et al., 93% survival for 1.5 million teeth at 5 years in Taiwan (55, 56, 61). Salehrabi and Rotstein, also studied nonsurgical retreatment with 89% survival for 4,744 teeth at 5 years in the USA (62).

Complications also have obvious impact on patients. Complications following RCT may include slow or inadequate healing, new disease, symptoms, swelling, tooth fracture, extraction, caries and periodontal disease. However, the complication and additional intervention rates for RCT are low, several times lower than for single tooth implants (63-68).

Teeth with RCTs have remarkably high long-term survival rates as measured longitudinally. However, cross-sectional data of the prevalence of disease in community populations presents a less positive picture; periradicular disease in both treated and untreated teeth is surprisingly common and technical quality of community RCT is broadly decried (69); room for improvement remains.

### 2.5. Economics

Cost is a significant barrier to receiving care for toothache and a very important factor in patients’ treatment choices (38). Initial cost may grab patients’ attention, but that is only the beginning. The initial cost of tooth retention through RCT and restoration is considerably lower than tooth replacement using implants or fixed dental prostheses (70-73). Lifetime costs may be more important; it appears that teeth retained through RCT have fewer complications than replacements using single tooth implants or fixed dental prostheses (63-68). A simple lifetime cost model, including treatment failures, ranked all pathways beginning with RCT as being less costly than all options beginning with implant treatment (74). Cost effectiveness analyses compare relative costs and outcomes, but do not monetize patient value. A cost effectiveness modeling study from the United Kingdom regarding a maxillary incisor reported that RCT was a highly effective first line intervention; nonsurgical retreatment was also cost effective; surgical retreatment was not cost effective; and that implants may have a role if nonsurgical retreatment fails (74). An American cost effectiveness modeling study, for a failed endodontically treated molar, ranked endodontic microsurgery, nonsurgical retreatment, replacement using a fixed dental prosthesis, and replacement using an implant, from most to least cost effective (75). A Canadian cost utility analysis, relating direct cost to the perceived change in quality of life, ranked removable partial dentures, RCT and restoration, fixed dental prostheses, in order from most to least efficient service (76). The same study ranked cost benefit, including monetized value, in the same order (76). Tooth position and amount of insurance coverage influence patient ranking of cost utility and cost benefit (76). Although, the removable partial denture had the highest cost utility and cost benefit rankings, it was the least preferred choice. Loss of a maxillary incisor was not tolerated, but loss of a less visible mandibular molar was tolerable; patients were willing to pay more out of pocket to save an anterior tooth (76). Cost is not everything, the key factor for dictating treatment should be prognosis for the remaining tooth (70, 71). The health care economist will tell us that all things being equal, the alternative to the natural state must be either somehow better or less costly; that is, the natural tooth has intrinsic value (72).

## 3. Discussion

The social impact, or quality of life impact, of RCT was investigated by several authors who used shortened versions of the OHIP (7, 10-12). The OHIP provides both global and detailed assessment of functional limitation, physical pain, psychological discomfort, physical disability, psychological disability and social disability. Two different short versions of the OHIP were used; some authors used it before treatment, others at differing intervals after treatment. Considerable congruence in findings was found amongst these different studies. Provision of RCT resulted in broad improvement of quality of life, especially in physical pain, psychological discomfort, psychological disability, and social disability.

OHIP metrics are not the only outcomes valued by patients. Anxiety, pain and cost appear to be of preeminent concern to patients facing the choice among RCT and tooth retention, tooth extraction without replacement, and tooth extraction with replacement. The pain and economic data reviewed in this paper are extremely supportive of the RCT option. However, people possess complex sets of emotions, beliefs, behaviors and values. Anxiety and fear play important roles in our behaviors and remembered experiences. Appearance is also of tremendous importance, patients place a high value on the appearance of their anterior teeth (11, 76). Dentists must understand the larger psychosocial environment as well as the technicalities of diagnosis, treatment and follow up.

Success was not included as a theme in this narrative review because differing criteria produce different results; furthermore most success/failure instruments are radiograph-based, not patient-centered. It is important to note that radiographically evident periapical disease, a usual determinant of failure, is often not painful or symptomatic. The authors suggest that comprehensive new endodontic outcome assessment indices, including patient-centered metrics, should be developed.

Previous narrative and systematic reviews have contrasted RCT with its alternatives, notably implants (57, 77). Likewise, others have previously reviewed treatment decision making (78), whereas, this paper focused upon the distinctive and unique features of patient-centered endodontic outcomes.

Patient education is critically important. Patients disproportionally fear RCT. However, compared to its alternatives such as extraction and replacement, RCT is less invasive, less costly, less time consuming, with low levels of intraoperative pain, and causes a greater reduction in pain felt by patients. Acceptance of this knowledge can reduce anxiety, fear, experienced, and remembered pain.

## 4. Conclusions

1.Disease of pulpal origin affects quality of life, with moderate severity, primarily through physical pain and psychological discomfort; RCT results in broad improvement of quality of life.

2.Satisfaction with RCT is extremely high; cost is the primary reason for dissatisfaction.

3.Anxiety and fear affect RCT patients, profoundly influencing their behaviors, including treatment avoidance, and felt pain.

4.Pain is widely feared, disliked, and remembered; RCT causes a dramatic decrease in pain prevalence and severity over the week following treatment.

5.Survival rates of teeth after RCT are very high; complication rates are low.

6.Cost is a barrier to RCT, but initial costs, lifetime costs, cost effectiveness, cost utility, and cost benefit all compare extremely well to the alternatives of extraction and replacement using implants or fixed prostheses.
